# Determinants of delayed healthcare seeking among STI patients in Eastern Ethiopia: a case-control study

**DOI:** 10.3389/fpubh.2025.1522882

**Published:** 2025-10-23

**Authors:** Dereje Bifa, Delelegn Yilma, Tsegaye Banti, Lechisa Asefa

**Affiliations:** ^1^Department of Public Health, Ambo University, Ambo, Ethiopia; ^2^Department of Environmental Health, Bule Hora University, Bule Hora, Ethiopia

**Keywords:** delay, STI, patients, Ethiopia, behavior

## Abstract

**Background:**

Delayed healthcare seeking is one of the major impediments to successfully preventing and controlling sexually transmitted infections. A delay in healthcare seeking prolongs the period of infectiousness and increases the incidence of sexually transmitted diseases. The majority of sexually transmitted infections are curable, but several social and behavioral factors hinder people from seeking early healthcare.

**Objective:**

To identify determinants of delay in healthcare seeking among patients with sexually transmitted infections at governmental hospitals in the Harari region, Ethiopia.

**Methods:**

In this study, a facility-based, unmatched case-control study was conducted. A total of 197 cases and 197 control study participants were selected by a systematic random sampling technique from patients with sexually transmitted infections. Data were collected through face-to-face interviews using a pre-tested structured questionnaire. Collected data were entered using Epi-Data version 4.6 and then exported to SPSS 26.0 for analysis. Bivariable analysis was done to select candidate variables for multivariable analysis at a p-value less than 0.25. Finally, multivariable logistic regression analysis was performed to identify determinants of delay in healthcare seeking based on AOR, with 95% CI and a p-value less than 0.05. Model fitness was checked by the Hosmer and Lemeshow test, and multicollinearity was checked with the variance inflation factor.

**Results:**

The current study identified that small family size (AOR = 0.2; 95% CI:0.14–0.73), unmarried marital status (AOR = 2.6; 95% CI: 1.02–18.24), poor knowledge (AOR = 3.26; 95% CI: 1.06–9.97), fear of social stigma (AOR = 4.75; 95% CI:1.4–9.97), having a single sexual partner (AOR = 0.31; 95%CI: 0.10–0.95), and non-enrolled in CBHI (AOR = 2.31; 95%CI:1.11–9.45) were found to be determinants of delay in healthcare seeking.

**Conclusion and recommendation:**

This study revealed that having a large family size, unmarried marital status, poor knowledge, fear of social stigma, number of sexual partners, and being non-enrolled in community-based health insurance were determinants of delay in healthcare seeking among patients with sexually transmitted infections. It was recommended that health education and awareness creation should be done for STI patients so that they become members of community-based health insurance.

## 1 Background

According to the World Health Organization (WHO), a sexually transmitted infection (STI) is defined as an infection caused by bacteria, viruses, or parasites that can be transmitted from one person to another through sex or intimate contact. Currently, there are over 30 pathogens, including bacterial, viral, and parasitic, that can cause STIs (1). Healthcare-seeking behavior is referred to as an action undertaken by individuals who perceive themselves as having a health problem or are ill to identify the best course of action (2).

It is a complex interaction of factors that involves the time between the onset of disease and seeking medical care, the type of medical care chosen, and the reasons for that choice, as well as medication compliance (3). The activities that people do when they have symptoms or suspect they have an STI have a big impact on disease transmission and control. Poor health outcomes, higher rates of morbidity and mortality, and unfavorable health statistics have all been associated with inappropriate healthcare-seeking behavior (4).

Sexually transmitted infections are a public health issue that hurts people’s quality of life and leads to significant morbidity and mortality (5). Around 1 million curable STIs are diagnosed every day around the world. According to WHO estimates, in 2016, 376 million new infections of the four curable STIs, such as chlamydia, gonorrhea, syphilis, and trichomoniasis, occurred (6). Furthermore, adolescents and young adults have the highest incidence of treatable STIs, with up to one in every 20 teenagers developing a new STI each year (7). STIs are an international issue; however, there are geographical differences. STIs are exceedingly common in developing countries, approximately 108 million STIs occur annually. It is believed that low-income nations account for 80 to 90 percent of the global burden of STIs (6).

Early diagnosis and treatment are central issues in the control and prevention of STIs (8). However, the prevalence of delayed treatment for STIs in sub-Saharan African countries ranges from 41 to 61%, which is very high (9). In Ethiopia, a study conducted in the Gambela region and Benishangul Gumuz region showed that 56.8% (10) and 59.9% (11) of patients with STIs were delayed in healthcare seeking, respectively. The majority of STIs are curable, but several social and behavioral factors hinder people from seeking healthcare early, which increases the burden of the disease (11). Socio-demographics, cultural, self-medicating behavior, physical and financial affordability of health services, poor knowledge about STIs, and low socioeconomic status are the risk factors for delayed healthcare seeking for STIs (2, 12).

Delayed treatment or an untreated infection could lead to harsh conditions, such as inflammation of the cervix, urethritis, and ulceration of the genitals, pelvic inflammatory diseases, ectopic pregnancy, infertility, cardiovascular diseases, blindness, severe or long-term disability in infants, and finally death (13). The majority of the complications of STIs are preventable if the patient is diagnosed and treated early (14). Untreated STIs in developing countries account for about 17% of all economic losses (9).

A thorough understanding of the healthcare-seeking behavior of patients with STIs is very necessary for the implementation of STI control programs. Early healthcare utilization and adherence to effective treatment can reduce morbidity, disability, and mortality resulting from STIs. In contrast, a delay in healthcare seeking prolongs the period of STI and thereby increases the incidence of the infection of STIs, including HIV (11). Factors that delay healthcare seeking and prolong the period of infectiousness of STI are of great clinical and public health importance. Timely healthcare-seeking behavior is essential for controlling sexually transmitted infections (STIs). Delays in seeking care extend the infectious period and contribute to increased STI incidence. Despite the curability of most STIs, social and behavioral factors hinder early treatment. However, there is a limited study about the factors that determine being late for healthcare seeking among patients with STIs. Therefore, the study’s objective was to identify factors contributing to delays in healthcare-seeking among STI patients in government hospitals in the Harar Region, Ethiopia.

## 2 Methods

### 2.1 Study area and period

This study was conducted in the Harari region. The Harari region is one of the 11 regional states located in the eastern part of Ethiopia at a distance of 525 km from Addis Ababa. The region is the smallest in terms of population size and area. The region has a total estimated area of 311.25 square kilometers and an estimated density of 589.05 people per square kilometer. It is also one of the most urbanized regions, with 54.18% of its population residing in urban areas.

### 2.2 Study design

An institution-based, unmatched case-control study design was used.

### 2.3 Population

#### 2.3.1 Source population

All patients with STIs at governmental hospitals in the Harar region.

#### 2.3.2 Study population

The study population consisted of cases and control groups. The cases were patients who were newly diagnosed as having an STI and sought treatment in the hospitals after 7 days of developing the first signs/symptoms during the study period. Controls were patients who were newly diagnosed as having an STI and sought treatment in the hospitals within 7 days of developing the first signs/symptoms during the study period.

### 2.4 Inclusion and exclusion criteria

#### 2.4.1 Inclusion criteria

Patients who were newly diagnosed as having STIs during the study period.

#### 2.4.2 Exclusion criteria

Patients with STIs who failed to mention the exact date of onset of symptoms were below 15 years of age, and mentally ill patients were excluded from the study.

### 2.5 Sample size determination

A double population proportion formula was employed to determine sample size using Epi Info version 7 for sample size estimation for an unmatched case-control study design. The listed variable number of sexual partners was used to estimate the optimum sample size for this study (10). Assuming a 95% level of confidence, 80% power, 1:1 case to control ratio, the total sample size was 398 (199 cases and 199 controls), including 10% contingency for non-response rate.

### 2.6 Sampling technique and procedure

**Figure 1:**
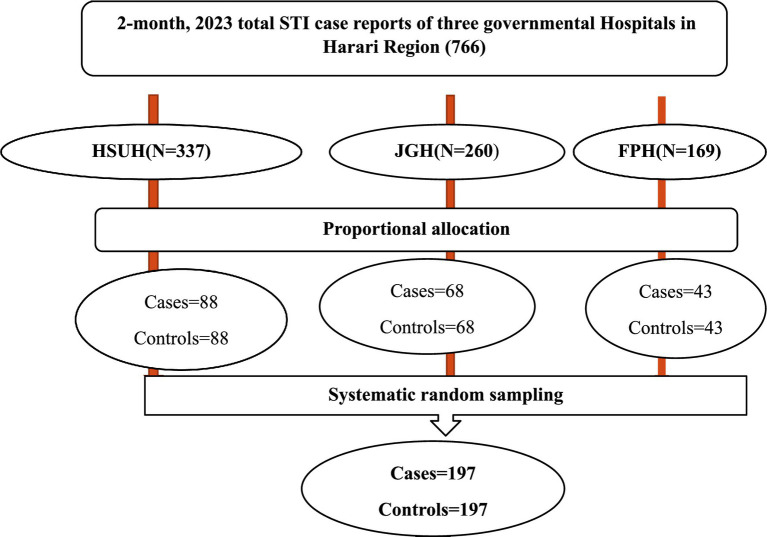
Sampling procedure for a study conducted on determinants of delay in healthcare seeking among patients with STIs at governmental hospitals in Harar Region, Eastern Ethiopia, 2023.

All three governmental hospitals in the region were selected for the study. These hospitals are Hiwot Fana Specialized University Hospital, Jugal General Hospital, and Federal Police Hospital. The sample size was allocated proportionally based on the number of STI patients reported from each hospital during October and November 2023. Finally, a systematic random sampling technique was used to select study participants with a K interval (K = N/n = 766/398 = 2; see Figure 1 for details).

### 2.7 Data collection tool and procedures

A structured, interviewer-administered questionnaire was used to collect data. The questionnaire was developed by reviewing different kinds of literature and existing guidelines, and finally modified for the context of the study. The questionnaire consisted of socio-demographic characteristics, knowledge about STIs, perceptions toward healthcare seeking, risky sexual behavior, substance use characteristics, type of syndrome, socio-cultural related characteristics, health facilities, and healthcare provider characteristics. The tool was developed in English and translated into Afan Oromo, as the majority of the population speaks Afan Oromo as a common language. Initially, systematically selected patients with STIs were verbally consented and eligible study participants from the Outpatient Department were linked to the data collectors, and the interview was conducted in a private room after patients received routine care. Facility: One supervisor (1 general practitioner) and three data collectors (BSc nurses) participated and trained for 2 days about research objectives, confidentiality, interviewing techniques, and ethical issues.

### 2.8 Study variables

#### 2.8.1 Dependent variable

Delay in healthcare seeking for patients with STI.

#### 2.8.2 Independent variables

##### 2.8.2.1 Socio-demographic variables

Age, sex, family size, marital status, income, educational status, religion, occupation, residence, education of partner, occupation of partner, monthly income, and member of CBHI.

##### 2.8.2.2 Knowledge variables

Transmission, recognize symptoms, prevention, and complication.

##### 2.8.2.3 Perception of respondent variables

Perceived toward communicability, perceived curability, perceived toward seeking healthcare, screening STI, perceived severity, time to decision healthcare.

##### 2.8.2.4 Type of syndrome and healthcare-seeking behaviors variables

Type of syndrome diagnosed, reason not treated early, source of advice to seek healthcare, know HIV status, and fear of social stigma.

##### 2.8.2.5 Risky sexual behavior and substance variables

Age at first sex, number of sexual partners, use of condom, history of sex with FSW, sexual activity practice while symptomatic, history of chewing chat and drinking alcohol.

##### 2.8.2.6 Health facilities and healthcare provider variables

Distance, waiting time, privacy, cost of service, received need care, and interaction of healthcare provider.

### 2.9 Operational definition

#### 2.9.1 Cases (delay)

Patients who were newly diagnosed as having STI and sought treatment in the hospitals after 7 days of developing the first signs and symptoms (10, 15).

#### 2.9.2 Controls

Patients who are newly diagnosed as having STI and sought treatment in the hospitals within 7 days of developing the first signs and symptoms (10, 15).

#### 2.9.3 Knowledge about STIs

A mean score was used to determine the knowledge status of respondents on STIs. Respondents who scored above the mean were categorized as having good knowledge, and those who scored equal to or below the mean were categorized as having poor knowledge (10).

#### 2.9.4 Perception about STIs

A mean score was used to determine the perception of respondents on STIs. Respondents who scored above the mean were categorized as having a positive perception, and those who scored equal to or below the mean were categorized as having a negative perception (16).

### 2.10 Data quality control

Reliability/validity testing of the questionnaire was checked using Cronbach’s α statistic, which was 0.83. The data collection tool was first prepared in English and translated into Afan Oromo and then back-translated to English by a different person to ensure consistency. Data were collected by trained data collectors. Training was given to the data collectors on the objective of the study, the process of data collection, and field ethics for 2 days. A pretest of the instrument was conducted before the actual data collection on 5% of the study subjects in Haramaya General Hospital. The necessary modifications were made to the tool. The collected data were checked by the supervisor and the principal investigator for completeness and accuracy, and errors were corrected daily before leaving the hospitals.

### 2.11 Data processing and analysis

The data were checked for completeness, cleaned, coded, and entered using Epi-Data version 4.6 and then exported to SPSS version 26.0 for analysis. Descriptive statistics, such as percentages and frequencies, were carried out for both cases and control groups, and the results are presented in text, figures, and tables. A bivariate analysis was used to select candidate variables for multivariable analysis. Each variable with an outcome of interest at a *p* value < 0.25 in the bivariate analysis was a candidate for multivariable analysis to control the possible effect of confounders. Finally, multivariable logistic regression analysis was performed to identify determinants for the outcome variable based on AOR, with a 95% CI and a *p*-value less than 0.05. Model fitness was checked by the Hosmer and Lemeshow goodness test, and it was 0.877, and it was also checked with a variance inflation factor.

## 3 Results

### 3.1 Socio-demographic characteristics

A total of 394 respondents (197 cases and 197 controls) were involved in this study, with a response rate of 98.9%. More than half of the cases, 106 (53.8%), and controls, 109 (55.3%), were within the age group of 25–34 years. About 137 (69.5%) of cases and 166 (84.3%) of controls were living in rural areas. In most cases, 91 (46.2%), and controls, 103 (52.3%), were married. About 130 (66%) of cases had a family size greater than five. More than half, 113 (57.3%), of cases were not enrolled in community-based health insurance. The majority of cases followed primary education 76 (38.5%; Table 1).

**Table 1:** Socio-demographic characteristics of patients with STI at Governmental Hospitals of Harar Region, Eastern Ethiopia, 2023.

Variable	Response	Cases number (%)	Controls number (%)
Age	15–24	69 (35)	58 (29.4)
	25–34	106 (53.8)	109 (55.3)
	>35	22 (11.2)	30 (15.2)
Sex	Male	96 (48.7)	122 (61.9)
	Female	101 (51.3)	75 (39.1)
Family size	≤ 5	67 (34%)	152 (77.2%)
	>5	130 (66%)	45 (22.8%)
Marital status	Single	81 (41.1)	57 (28.9)
	Divorced	4 (2)	21 (10.6)
	Widowed	21 (10.6)	16 (8.1)
	Married	91 (46.2)	103 (52.3)
Religion	Muslim	111 (56.3)	89 (45.2)
	Orthodox	58 (29.4)	65 (33)
	Protestant	24 (12.2)	38 (19.3)
	Wakefata	4 (2)	5 (2.5)
Place of residence	Rural	137 (69.5)	166 (84.3)
	Urban	60 (30.5)	31 (16.7)
Member of CBHI	Non-enrolled	113 (57.3)	71 (36)
	Enrolled	84 (42.6)	126 (64)
Educational statuses of respondents	Non-formal education	27 (13.7)	11 (5.5)
	Primary education	76 (38.5)	46 (23.3)
	Secondary education	61 (30.96)	85 (43.1)
	Diploma and above	33 (16.8)	55 (27.9)

### 3.2 Knowledge of respondents about STI

Of the total respondents, 82 (41.6%) of cases and 154 (78.1%) of controls had good knowledge about STIs, respectively. In the majority of cases, 196 (99.5%), and controls, 192 (97.5%), know HIV/AIDS as a type of STI. The study revealed that in almost all cases and in the control group, having sexual contact with multiple partners was a risk for STI transmission (Table 2).

**Table 2:** Knowledge of respondents about STIs at Governmental Hospitals of Harar Region, Eastern Ethiopia 2023.

Variable	Response	Cases number (%)	Controls number (%)
What is STIs	Infections transmitted by sexual intercourse	163 (82.7)	188 (95.4)
	Do not know	34 (17.3)	9 (4.6)
Which type of STIs did you know	Gonorrhea	107 (54.3)	158 (80.2)
	*Chlamydia trachomatis*	87 (44.2)	135 (68.5)
	Syphilis	122 (61.9)	153 (77.7)
	Chancriod	76 (38.6)	106 (53.8)
	*Lymphogranuloma venerum*	24 (12.2)	37 (18.8)
	Trichomoniasis	39 (19.8)	66 (33.5)
	HIV/AIDS	196 (99.5)	192 (97.5)
Which risks of STIs transmission did you know	Unprotected sex	175 (88.8)	189 (95.9)
	A condom tears during intercourse	152 (77.2)	184 (93.4)
	Having sexual contact with multiple partners	193 (98)	194 (98.5)
	Sharing sharp material	163 (82.7)	184 (93.4)
	Mother to child	91 (46.2)	147 (74.6)
Which prevention methods of STIs did you know	Abstinence	124 (62.9)	163 (82.7)
	Reduce number of sex partners	194 (98.5)	196 (99.5)
	Use of condoms	170 (86.3)	188 (95.4)
	Not sharing sharp material each other’s	176 (89.3)	189 (95.9)
What are the complications of untreated STIs	Upper genital tract infections	119 (60.4)	162 (82.2)
	Infertility	92 (46.7)	135 (68.5)
	Cervical cancer	60 (30.5)	97 (49.2)
	Enhanced transmission and acquisition of HIV	66 (33.5)	103 (52.3)
	Ectopic pregnancy	79 (40.1)	123 (62.4)
Knowledge	Good	82 (41.6)	154 (78.1)
	Poor	115 (58.4)	43 (21.9)

**Figure 2:**
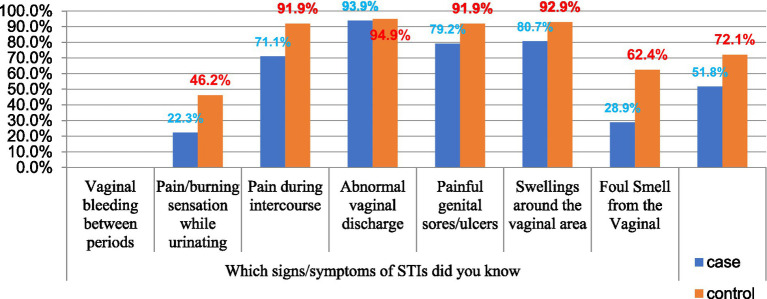
The signs of STI that are known by patients with STI at Governmental Hospitals of Harar Region, Eastern Ethiopia 2023.

From all participants, 93.9% of cases and 94.9% of controls said abnormal vaginal discharge is the sign/symptom of STI, and 22.3% of cases and 46.2% of the control group reported that burning sensation while urination is a sign of STI (see Figure 2).

### 3.3 Perceptions of respondents towards STIs

Of the study participants, 62 (31.4%) of cases and 78 (39.5%) of the control group had positive perceptions about STI. However, 87 (44.2%) of the cases and 53 (26.9%) of the control group did not believe screening for STI was important. 84 (42.6%) of cases and 80 (40.6%) of controls did not think getting an STI was serious to their health. 92 (46.7%) of cases and 86 (43.7%) controls decided to seek medical help at moderate signs of illness (Table 3).

**Table 3:** Perception of respondents toward STIs at Governmental Hospitals of Harar Region, Eastern Ethiopia 2023.

Variable	Response	Cases number (%)	Controls number (%)
Do you think STI is a communicable disease	Yes	174 (88.3)	190 (96.4)
	No	23 (11.7)	7 (3.6)
Do you think screening for STIs is good	Yes	110 (55.8)	144 (73.1)
	No	87 (44.2)	53 (26.9)
Do you think getting an STI is serious to your health	Not serious	84 (42.6)	80 (40.6)
	Neutral	36 (18.3)	20 (10.2)
	Very serious	77 (39.1)	97 (49.2)
Do you think a person who is infected with STI must be treated	Yes	107 (54.3)	139 (70.6)
	No	90 (45.7)	58 (29.4)
Can STDs be cured	Yes	178 (90.4)	181 (91.9)
	No	19 (9.1)	16 (8.1)
At what stage of illness did you decide to seek medical help	Mild	36 (18.3)	86 (43.7)
	Moderate	92 (46.7)	86 (43.7)
	Severe	69 (35)	25 (12.7)
Overall perception	Positive	62 (31.4)	78 (39.5)
	Negative	135 (68.5)	119 (60.4)

### 3.4 Socio-cultural characteristics

Most of the cases and controls were diagnosed with vaginal discharge, 48 (24.4%), and genital ulcers, 57 (28.9%), respectively. Of the total cases, 41 (20.8%) were not seeking treatment immediately after the onset of symptoms, hoping symptoms would go away. Respecting decision-making for health seeking, 115 (58.4%) of cases and 131 (66.5%) of controls decided by themselves. The majority, 143 (72.6%), of the cases had fear of stigma on experiencing symptoms of an STI (Table 4).

**Table 4:** Type of syndrome and healthcare seeking of patients with STI at Governmental Hospitals of Harar Region, Eastern Ethiopia 2023.

Variable	Response	Cases, number (%)	Controls number (%)
STI syndrome diagnosed by a healthcare provider	Genital ulcer	36 (18.3)	57 (28.9)
	Urethral discharge	31 (15.7)	41 (20.8)
	Vaginal discharge	48 (24.4)	39 (19.8)
	Inguinal bubo	27 (13.7)	5 (2.5)
	Itching	19 (9.6)	10 (5.1)
	Scrotal swelling	10 (5.1)	12 (6.1)
	Lower abdominal pain	26 (13.2)	33 (16.8)
Factors that prevented from seeking treatment immediately after the onset of symptoms	Went to pharmacy	39 (19.8)	
	Embarrassment/Stigma	39 (19.8)	
	Financial constraints	26 (13.2)	
	Hoping symptoms will go away	41 (20.8)	
	Didn’t have time	34 (17.3)	
	Distance to health facility	18 (9.1)	
Who decided for you to seek care for an STI	Myself	115 (58.4)	131 (66.5)
	Parents	18 (9.1)	17 (8.6)
	sexual partners	36 (18.3)	24 (12.2)
	Friends	28 (14.2)	25 (12.7)
Do you feel a fear of stigma on experiencing symptoms of an STIs	Yes	143 (72.6)	101 (51.3)
	No	54 (27.4)	96 (48.7)
Knowing HIV Status	Yes	64 (32.5)	98 (49.7)
	No	133 (67.5)	99 (50.3)

### 3.5 Risk sexual behavioral and substance use characteristics

Of the total respondents, 26 (36.1%) and 27 (38.6%) of controls and cases used condoms sometimes, respectively. In the majority of controls, 120 (60.9%) had single sexual partners, but in more than half of cases, 103 (52.3%) had more than two sexual partners. Of the total respondents, 34 (17.3%) of cases and 11 (5.6%) of controls had sex with female sex workers (Table 5).

**Table 5:** Risky sexual behavioral characteristics of respondents at Governmental Hospitals of Harar Region, Eastern Ethiopia 2023.

Variable	Response	Cases number (%)	Controls number (%)
Age at first sex	15–24	115 (58.4)	98 (49.7)
	25–34	82 (41.6)	99 (50.3)
Condom used during sex	Yes	70 (35.5)	72 (36.5)
	No	127 (64.5)	125 (63.5)
How often do you use	Always	11 (15.7)	17 (23.6)
	Usually,	32 (45.7)	29 (40.3)
	Sometimes	27 (38.6)	26 (36.1)
Number of sexual partners in the last 12 months	Single	94 (47.7)	120 (60.9)
	Two or above	103 (52.3)	77 (39.1)
Sexual activity practice while symptomatic	Yes	119 (60.4)	91 (46.2)
	No	78 (39.6)	106 (53.8)
Sexual practice with female sex worker	Yes	34 (17.3)	11 (5.6)
	No	163 (82.7)	186 (94.4)
Did you chew a chat	Yes	129 (65.5)	103 (52.3)
	No	68 (34.5)	94 (47.7)
Did you drink alcohol	Yes	89 (45.2)	80 (40.6)
	No	108 (54.8)	117 (59.4)

### 3.6 Health facilities and healthcare provider characteristics

Of the total respondents, 47 (23.4%) of cases and 16 (8.1%) of controls reported that the cost of treatment for healthcare was very difficult to pay. Of the total number of respondents, 144 (73.1%) of cases and 156 (79.7%) of controls reported that healthcare providers kept their privacy and confidentiality. Among respondents, 120 (60.9%) of cases and 157 (79.7%) of controls said that healthcare providers listen to their concerns/opinions during visits to health facilities (Table 6).

**Table 6:** Health facilities and healthcare provider care characteristic at governmental hospitals of Harar Region, Eastern Ethiopia 2023.

Variable	Response	Cases number (%)	Controls number (%)
Cost of treatment to seek healthcare	Easy to pay	60 (30.5)	95 (48.2)
	Difficult to pay	90 (45.7)	86 (43.7)
	Very difficult to pay	47 (23.9)	16 (8.1)
Interaction of HCP to seek healthcare	Caring and respectful	125 (63.5)	161 (81.7)
	Unfriendly and rude	72 (36.5)	36 (18.3)
The HCP keep your privacy and confidentiality	Yes	144 (73.1)	156 (79.7)
	No	53 (26.9)	41 (20.8)
HCP listens to your concerns/opinions during the visited HF	Yes	120 (60.9)	157 (79.7)
	No	77 (39.1)	40 (20.3)
HCP judgmental on the infection you had during last your visited healthcare	Yes	155 (78.7)	181 (91.9)
	No	42 (21.3)	16 (8.1)
How many hours take to reach HCF	>30 min	20 (10.1)	17 (8.6)
	< 30 min	177 (89.9)	180 (91.3)

### 3.7 Determinants of delay in healthcare seeking among patients with STI

To identify candidate variables for multivariate logistic regression, the association between each independent variable and the delay in healthcare seeking of patients with STI was done using binary logistic regression. Accordingly, sex, family size, marital status, educational status of respondents, place of residence, CBHI, knowledge, fear of social stigma, educational status of partner, number of sexual partners in the last 12 months, sexual practice with a female sex worker, and healthcare provider judgment on the infection you had during your last visit to healthcare had a significant association with delay in healthcare seeking among patients with STI at a p-value less than 0.25.

In multivariable logistic regression analysis, six variables were found to be significant predictors for the delay in healthcare seeking of patients with STI after controlling for possible confounders. Accordingly, STI patients with a family size of less than five were 80% less likely to delay health seeking compared to those who had greater than five (AOR = 0.2; 95% CL: 0.4–0.73). Those unmarried respondents were 2.6 times more likely to delay healthcare seeking compared to the married respondents (AOR = 2.6; 95% CI: 1.02–18.24). Respondents who had poor knowledge were 3.26 times more likely to delay healthcare-seeking compared to those who had good knowledge about STI (AOR = 3.26; 95% CI: 1.06–9.97). Patients with STI who had a fear of social stigma were 4.75 times more likely to delay seeking healthcare compared to those who had no fear of social stigma (AOR = 4.75; 95% CI: 1.4–16.02). Health-seeking patients with STI who had a single sexual partner were 69% less likely to delay in seeking healthcare compared to those who had two or more sexual partners (AOR = 0.31; 95% CI: 0.102–0.954). In addition, patients with STI who were not enrolled in community-based insurance were 2.31 times more likely to delay healthcare seeking compared to those who were enrolled in CBHI (AOR = 2.31; 95% CI: 1.11–9.45; Table 7).

**Table 7:** Bivariable and multivariable analyses using binary logistic regression model for determinants of delay in healthcare seeking among patients with STI at Governmental Hospitals of Harar Region, Eastern Ethiopia 2023.

Variable	Response	Patient categories		COR	AOR	*p* value
		Cases (Yes)	Controls (No)			
Sex	Male	96 (48.7%)	122 (61.9%)	0.58 (0.39 0.87)	0.61 (0.19–1.86)	0.275
	Female	101 (51.3%)	75 (39.1%)	1	1	
Family size	≤5	67 (34%)	152 (77.2%)	0.15 (0.11–0.58)	0.2 (0.14–0.73) *	0.000
	>5	130 (66%)	45 (22.8%)	1	1	
Marital status	Unmarried	106 (53.8%)	94 (77.6%)	1.27 (1.14–23.6)	2.6 (1.02–18.24) *	0.001
	Married	91 (46.2%)	103 (52.3%)	1	1	
Educational status of respondents	No formal education	27 (13.7%)	11 (5.6%)	4.09 (1.79–9.32)	0.41 (0.06–2.83)	0.125
	Primary school	76 (38.6%)	46 (23.4%)	2.75 (1.56–4.84)	0.86 (0.21–3.44)	0.548
	Secondary school	61 (31%)	85 (43.1%)	1.19 (0.69–2.06)	0.404 (0.12–1.39)	0.104
	Diploma and above	33 (16.8%)	55 (27.9%)	1	1	
Place of residence	Rural	137 (69.5%)	166 (84.3%)	0.43 (0.26–0.69)	1.73 (0.42–7.07)	0.81
	Urban	60 (30.5%)	31 (16.7%)	1	1	
CBHI	Non-enrolled	113 (57.4%)	71 (36%)	2.38 (1.59–12.81)	2.31 (1.11–9.45) *	0.002
	Enrolled	84 (42.6%)	126 (64%)	1	1	
Knowledge	Poor	115 (58.4%)	43 (21.3%)	5.02 (1.16–12.58)	3.26 (1.06–9.97) *	0.002
	Good	82 (42.6%)	154 (78.7%)	1	1	
Feel fear of social stigma	Yes	143 (72.6%)	101 (51.3%)	2.52 (1.65–3.83)	4.75 (1.4–16.02) *	0.012
	No	54 (27.4%)	96 (48.7%)	1		
Educational status of partners	No formal education	30 (33%)	13 (12.6%)	4.23 (1.62–11.03)	8.5 (0.78–91.77)	0.141
	Primary school	34 (37.4%)	37 (35.9%)	1.68 (0.72–3.92)	4.78 (0.54–41.93)	0.101
	Secondary school	15 (16.5%)	31 (30.1%)	0.88 (0.35–2.26)	1.6 (0.22–11.48)	0.786
	Diploma and above	12 (13.2%)	22 (21.4%)	1	1	
Number of sexual partners in the last 12 months	Single	94 (47.7%)	120 (60.9%)	0.58 (0.39–0.87)	0.31 (0.11–0.95) *	0.014
	Two or above	103 (52.3%)	77 (39.1%)	1	1	
Sexual practice with female sex worker	Yes	34 (17.3%)	11 (5.6%)	3.52 (1.73–7.18)	5.3 (0.09–25.43)	0.314
	No	163 (82.7%)	186 (94.4%)	1	1	
HCP judgmental on the infection you had during last visited healthcare	Yes	155 (78.7%)	181 (91.9%)	0.33 (0.176–0.603)	0.29 (0.05–1.72)	0.489
	No	42 (21.3%)	16 (8.1%)	1	1	

* indicates statistically significant association with outcome variable.

## 4 Discussion

Early seeking of healthcare treatment is very important for early diagnosis and treatment of diseases and for reducing the burden of disease (8). However, different literature shows there is a delay in seeking healthcare for sexually transmitted diseases (9, 10). Thus, this study identified the determinants of delay in healthcare seeking among patients with sexually transmitted infections.

Community-based health insurance played a great role in early treatment seeking; patients with STI who were not enrolled in community-based health insurance were 2.31 times more likely to be delayed in healthcare seeking as compared to those who were enrolled in community-based health insurance. In support of this, a study conducted in Sub-Saharan Africa reveals that non-enrolled community-based insurance has a significant impact on the health-seeking of patients (17). This might be due to out-of-pocket payments for healthcare services, which may hinder early healthcare utilization among patients. Thus, it encouraged the community to be a member of CBI by the local government.

Participants who reported fear of stigma for being exposed to STIs were 4.75 times more likely to delay seeking healthcare than those who had no fear of social stigma. This finding is consistent with the studies done in southwest Oromia (18), Gambela (10), Nkomazi East, and South Africa (19). Seeking treatment in a timely manner may still be hampered by the stigma associated with STIs. For instance, patients with STIs often feel stigmatized about seeking care for their STIs in health facilities where they are familiar with healthcare providers. The possible reason for this could be fear of the provider’s judgment and stigma, as well as social embarrassment (20).

In the current study, the delay in healthcare seeking for STIs was 3.26 times more likely among those who had poor knowledge of STIs compared to those who had good knowledge. This finding is consistent with the study conducted in Gambela (10), southwest Oromia (10), southwest Oromia (18), and Sub-Saharan Africa (9). This indicates that those who have better knowledge of STIs play a role in reducing the burden of the disease by shortening the duration of infection and spreading it through early healthcare seeking. Closing the knowledge gap regarding STIs through education and counseling is a strategy to avoid the delay in healthcare seeking and prevent the development of complications from STIs (9, 17, 21). In order to reduce the delay in health-seeking, implementation of a health education program set as policy in a health institution is very pertinent.

Additionally, it was shown that a patient’s marital status was another factor that was statistically linked to the delay in seeking medical attention among STI patients. Accordingly, respondents whose marital status was unmarried were 2.6 times more likely to delay seeking healthcare for STIs compared to married respondents. This finding was consistent with studies conducted in Bangladesh (22), south-west Ethiopia (23), and south-west Oromia (18), which found that married STI patients were more likely to seek early treatment. This could be because married individuals who are in a relationship with their husband or wife might be influenced to seek treatment early since there will be strong communication among them on the symptoms that were manifested among them. This suggests that non-married STI patients should be given priority in improving STI patients’ healthcare-seeking behavior. In contrast, studies conducted in Gambel, Ethiopia (12), Nkomazi East, and Mpumalanga (24) did not find marital status as factors that determine the health-seeking behavior of STI patients. This might be due to sample size and variation in sociodemographic characteristics of the respondents as well as the variables studied.

In this study, delay in health-seeking among patients with STI had a significant association with family size. Accordingly, respondents whose family size was less than five were 80% times less likely to delay in seeking healthcare compared to those who had greater than five. This finding was consistent with the study conducted in Sidama (25) and northwest Ethiopia (26). This might be because those who have larger family members carry more responsibilities and have experienced severe socio-economic hardship, which prevented them from early healthcare for the symptoms they experienced.

In addition, respondents who have single sexual partners were 69% less likely to delay seeking healthcare compared to those who have multiple sexual partners. This finding was consistent with a study conducted in Gonder (27) and Gambella (10). The possible explanation could be that those with a single partner are often married or in a union, and so they worry about their health as well as the health of their partner. In addition, fear of complications and being committed to risky sexual behavior may trigger them to seek care early (10, 19, 28).

Moreover, a study conducted in Bangladesh (22) shows a significant association between healthcare seeking of STI and age, sex, and perceived severity of STI, which was inconsistent with this current study. Furthermore, study conducted in northern Ethiopia (29) found that distance to health facilities and cost of treatment were determinant factors for healthcare seeking of STI patients, which were not found to be determinants of delay in healthcare seeking among patients with STI. This discrepancy might be due to socio-demographic characteristics and sample size.

### 4.1 Strengths and limitations of the study

Strengths of the study were that data were collected by health professionals, and a case-control study was used in this study. However, this study was conducted at hospitals, and the results of this study are less generalizable to the community. It is noted that this study includes only patients who experienced STI symptoms and sought care at hospitals. However, as patients who did not seek care might have different characteristics, generalizability should be done cautiously.

## 5 Conclusion

Delay in health-seeking of STI patients had a significant association with large family size, poor knowledge of the patient about STI, unmarried marital status, fear of stigma, non-enrollment in community-based health insurance, and having more than one sexual partner. It is recommended to create awareness about early healthcare seeking and encourage the community to be a member of the CBI.

## Data Availability

The raw data supporting the conclusions of this article will be made available by the authors, without undue reservation.
